# Comparison of Saliva Nitric Oxide between Chronic Kidney Disease Before and After Dialysis and with Control Group

**DOI:** 10.2174/1874210601812010213

**Published:** 2018-03-28

**Authors:** Fatemeh Rezaei, Reza Mohhamadi

**Affiliations:** 1 Department of Oral Medicine, School of Dentistry, Kermanshah University of Medical Sciences, Kermanshah, Iran; 2 School of Dentistry, Kermanshah University of Medical Sciences, Kermanshah, Iran

**Keywords:** Chronic kidney disease, Dialysis, Nitric Oxide, Saliva, Wilcoxon test, Glomerular Filtration Rate (GFR)

## Abstract

**Introduction:**

Chronic Kidney Disease (CKD) is a chronic progressive disorder and a major cause of death and disability in all countries. In the kidneys, Nitric Oxide (NO) has involved in several important cellular processes including glomerular and modular hemodynamics set-out, tubular - glomerular feedback reaction, renin releasing and extracellular fluid volume but NO can act as an inflammatory mediator and oxidative stress factor in high levels.

**Aim:**

The aim of this study was to evaluate salivary levels of NO in patients with chronic kidney disease on dialysis compared to the healthy subjects and evaluate the effect of dialysis on the level of NO in saliva.

**Materials & Methods:**

In this case-control study, 30 hemodialysis patients and 30 healthy controls that were matched for age and sex were selected. Unstimulated saliva samples were collected from all subjects. In the patient’s group, half an hour before starting dialysis first sampling and two hours after the completion of dialysis second sampling were collected. NO concentration in the samples was measured by using the Griess method. For data analysis, *SPSS* software version 16, Mann Whitney-U and Wilcoxon test were used. The level of significance was considered 0.05.

**Results:**

Mann-Whitney U test showed that the average concentration of salivary NO in patients with CKD (pre-dialysis and after dialysis) was higher than in the control group. The average concentration of salivary NO in patients with CKD was reduced after hemodialysis.

**Conclusion:**

Hemodialysis reduces salivary NO levels in CKD patients. It seems that hemodialysis has a role in decreasing the concentration of this inflammatory mediator and oxidative stress.

## 1 INTRODUCTION

Chronic Kidney Disease (CKD) is one of the major causes of morbidity and mortality globally. This is a progressive condition characterized by a reduction in Glomerular Filtration Rate (GFR), structural and functional disorders. Hypertension, diabetes, hyperlipidemia, obesity, and smoking are risk factors for CKD [1]. Cardiovascular diseases, diabetes and cancer are main consequences of CKD [2].

In oral cavity prevalence of oral diseases such as moderate periodontitis, gum bleeding, xerostomia, candidiasis, burning mouth and abnormal taste are higher in CKD patients compared with individuals without CKD [3, 4].

NO is a molecule that has several roles in physiologic and pathologic functions such as neural transmission, vasodilation, immunomodulation, myocyte contraction, inhibition of platelet aggregation, regulation of gene transcription, and mRNA translation [5]. NO can act as an antioxidant with protective effects against the injuries resulted by reactive oxygen species (ROS). At the same time, it can act as a free radical molecule in high levels. It converts to nitrite and nitrate and reacts with O_2_^-^ to form ONOO^-^ [6]. NO has the ability to kill bacteria, viruses, protozoans, and tumoral cells. However, NO has detrimental effects on proteins, DNA, and cellular lipids and can cause cell death, tissue injury, and decreased the function of various body systems [7].

In the kidneys, NO plays role in some important mechanisms such as regulation of glomerular and medullary hemodynamic, tubuloglomerular feedback, renin release, and regulation of extracellular fluid [8]. Some conditions which can occur in the renal system as the consequence of the rise in NO include glomerulonephritis, postischemic renal failure, radiocontrast nephropathy, obstructive nephropathy, and renal transplant rejection [9]. On the other hand, decreased NO production in patients with CKD can lead to salt retention and hypertension [10]. Several studies have shown that CKD patients are exposed to oxidative stress. This condition can cause high morbidity and mortality rate [11]. The imbalance between free radicals and antioxidants in saliva may play significant roles in the beginning and developing of oral diseases [12, 13]. Hemodialysis (HD) is the treatment for chronic renal failure patients, which has been successful in extending lifespan of renal patients and is effective in correcting the metabolic abnormalities related to renal oxidative stress that contributes to morbidity in CKD patients [11].

Therefore, the objective of the current study was first, to compare salivary NO level between CKD patients and control group and second, to define the effect of hemodialysis on salivary nitric oxide level.

## 2 MATERIALS AND METHODS

In this study, 30 CKD patients who were receiving hemodialysis for at least six months were recruited. These included patients were presenting to Imam Reza Hospital, Kermanshah, Iran (governmental and referral hospital). Thirty control subjects who were matched with the hemodialysis group regarding age and gender were also included. Inclusion criteria were age over 18 years, GFR < 15 mL/min, Blood Urea Nitrogen (BUN) > 20 mg/dl, serum creatinine > 3 mg/dl, and creatinine clearance < 20 mL/min. In the control group, the subjects were healthy in general. Exclusion criteria were periodontal conditions, kidney transplantation, and smoking cigarettes .

Unstimulated salivary sampling was done in both groups. The samples were asked not to eat or drink for two hours prior to saliva sampling, seat still, tilt head forward so that saliva accumulates in the anterior part of the mouth. Then, after swallowing the contents of the primary saliva, the re-accumulated saliva was collected in a tube [14]. In the hemodialysis group, the first sampling was done half an hour before hemodialysis initiation and the second sampling were performed two hours after hemodialysis termination [15]. The samples were centrifuged at 3,000 rpm for 10 minutes and the supernatant was analyzed. The samples were stored at - 80°C until the analysis time. The NO level was measured using Griess method [15].

The gathered data were entered into a checklist. The analyses were done using *SPSS* software (ver. 16.0). The normal distribution of the data was determined using the Kolmogorov-Smirnov (KS) test. In order to compare the data between hemodialysis and control groups, the Mann-Whitney U test was used. To compare the data before and after hemodialysis, the Wilcoxon test was applied

## 3 RESULTS

There were 18 males (60%) and 12 females (40%) in either group. Mean ± SD ages in hemodialysis and control groups were respectively 58.13 ± 9.61 and 60.77 ± 7.9 years.

The KS test showed that the data had not normal distribution (Table **1**).

Mean salivary NO concentrations in hemodialysis group before and after hemodialysis were respectively 584.31 ± 510.7 (μmol/L) and 281.62 ± 340.78 (μmol/L). This value in control group was 44.45 ± 26.89 (μmol/L). The Mann-Whitney test showed that a significant difference existed between control and pre-hemodialysis levels (*P*< 0.001). The median salivary NO level was lower in the control group as compared to the hemodialysis group.

The Mann-Whitney test also demonstrated that salivary NO level had a significant difference between control group and post-hemodialysis values (*P*< 0.001). The median salivary NO level was lower in the control group as compared to the hemodialysis group.

The Wilcoxon test showed that hemodialysis had a significant effect on salivary NO level (*P*< 0.001). The median salivary NO level was lower in post-hemodialysis state compared to pre-hemodialysis values (Table **2**).

## 4 DISCUSSION

The current study was conducted to compare salivary NO level between CKD patients on hemodialysis and healthy controls. The saliva is composed of the gingival crevicular fluid and secretions of the major and minor salivary glands. The constituent of gingival crevicular fluid is similar to serum [16]. CKD can change the saliva in quality and quantity [17, 18]. Several studies have been done regarding the role of salivary NO in oral diseases. Increased NO synthesis in periodontal diseases [19] and radicular cysts and apical infections [20], the role of NO in the pathogenesis of periodontitis and subsequent bone loss [21], due to inflammation, benign and malignant tumors of the salivary glands [22], squamous cell carcinoma [23] and angiogenesis and modulation of matrix metalloproteinase expression and decreased synthesis of natural inhibitors of cell synthesis [24] have been noted in the literature. NO in high concentration as an oxidative stress has a role in oral diseases such as lichen planus and periodontal diseases [25, 20].

According to the obtained results, the salivary NO level was significantly higher in CKD patients compared to control group. This is incompatible with the results reported by Clermont *et al.* who showed that plasma NO in CKD patients was higher than in control group. Which indicates the possible role of the interaction between blood and saliva [26]. Matsumoto *et al*. evaluated expiratory NO in CKD patients and related increased NO level with pathologic consequences [27]. It is because of inadequate renal clearance and renal function in the exertion of toxic and waste products from the blood.

In this study, salivary NO in CKD patients who were receiving hemodialysis was significantly higher compared to the control group. This value decreased significantly after hemodialysis. This shows that hemodialysis decreased salivary NO significantly. The results of two studies are in agreement with our results. A former study [28] showed that salivary NO level was lower in CKD patients who were receiving hemodialysis for four years compared to other patients. Another study reported that NO level decreased significantly after hemodialysis [29]. Many mechanisms are likely to be responsible, including substrate limitation, L-arginine is the precursor in the biosynthesis of NO. During hemodialysis process, L-arginine can be removed decreasing its concentration in the blood and impaired endothelial function may cause a decrease in NO level in post hemodialysis samples [29] and because of a direct correlation between serum and saliva, salivary NO decreased significantly after hemodialysis. However, our results contradict the results reported by Uzun *et al*. who reported decreased plasma NO in CKD patients and increased levels in hemodialysis patients [30]. Lao *et al*. reported that in End-Stage Renal Disease (ESRD) patients, NO production may increase due to induction of NO productive enzymes [31]. Other studies are in agreement with these results. For example, Blum *et al*. reported decreased NO level in CKD patients [32]. Other studies also reported decreased NO level in ESRD, hemodialysis, and peritoneal dialysis patients [33]. Nagane *et al*. reported that NO level was lower in renal failure patients compared to control group and this decreased level was associated with increased mortality due to cardiovascular diseases [34].

## CONCLUSION

The results of the current study showed that salivary nitric oxide level in CKD patients was higher than healthy controls. This level decreased after hemodialysis. As nitric oxide acts as an inflammatory mediator and oxidative stress factor in high levels, it seems that hemodialysis, by decreasing the concentration of this mediator, has a role in the reduction of oral lesions such as lichen planus and periodontal diseases.

## ETHICS APPROVAL AND CONSENT TO PARTICIPATE

This study was approved by the Research Deputy of Kermanshah University of Medical Science, Kermanshah, Iran (#98093).

## HUMAN AND ANIMAL RIGHTS

No animals were used in this research. All research procedures followed were in accordance with the ethical standards of the committee responsible for human experimentation (institutional and national), and with the Helsinki Declaration of 1975, as revised in 2008.

## CONSENT FOR PUBLICATION

Consent form was obtained from the patients

## Figures and Tables

**Table 1:** The hypothesis of normal distribution of data.

–	t	*P* value
Before Hemodialysis	0.178	0.016
After Hemodialysis	0.250	< 0.001
Control	0.296	< 0.001

**Table 2:** Salivary nitric oxide levels (μmol/L) in hemodialysis and control groups.

–	Before Hemodialysis	After Hemodialysis	Control	*P* value ^a^	*P* value ^b^	*P* value ^c^
No.	30	30	30	< 0.001	< 0.001	< 0.001
Mean(μmol/L)	584.31	281.62	44.45			
SD	510.7	340.78	26.89			
Minimum(μmol/L)	33.30	31.40	10.60			
Maximum(μmol/L)	1407	1321	128.30			
First quartile	135.60	41.70	28.50			
Median	419.50	114.50	38.20			
Third quartile	1066	432.10	42.5			

^a^ Comparison between control and pre-hemodialysis (Mann-Whitney U test) ^b^ Comparison between control and post-hemodialysis (Mann-Whitney U test) ^c^ Comparison between pre- and post-hemodialysis
